# Anterioposterior Views Coupled With Lateral Views Are the Best for the Intraoperative Radiographic Detection of Retained Surgical Sponges

**DOI:** 10.7759/cureus.63583

**Published:** 2024-07-01

**Authors:** Kedar P Padhye, Bayard C Carlson, John M Dawson, Abdul Fettah Buyuk, Amir A Mehbod

**Affiliations:** 1 Department of Orthopaedics, Izaak Walton Killam Health Centre, Halifax, CAN; 2 Orthopaedic Surgery, Twin Cities Spine Center, Minneapolis, USA; 3 Research, Twin Cities Spine Center, Minneapolis, USA

**Keywords:** intraoperative complications, radiography, imaging, lumbar spine surgery, surgical textile products, retained foreign bodies, surgical sponges

## Abstract

Introduction: A retained sponge after spine surgery can cause serious medical complications and medicolegal problems. Intraoperative radiographs are commonly used to detect it. This study evaluated intraoperative radiographs under routine clinical conditions that most spine surgeons experience to detect retained sponges.

Methods: In this prospective randomized clinical trial, two patient groups undergoing open posterior lumbar surgery were studied. In one, a sponge was intentionally present; in the other, none was present. Standard intraoperative lateral (LAT) and anteroposterior (AP) radiographs were acquired before closing. Radiographs were analyzed for sensitivity, specificity, inter- and intraobserver reliability for three viewing conditions: one LAT radiograph versus one AP radiograph versus one LAT and one AP X-ray (LAT+AP).

Results: A total of 111 patients were included. Accuracy, interobserver reliability, and intraobserver reliability were best for LAT+AP (80%, 96%, and 96%, respectively). Sensitivity was best for LAT+AP (87%) and specificity was best for LAT (95%). Positive predictive value was best for LAT (94%); negative predictive value was best for LAT+AP (88%). The probability of being right is better for female sex (odds ratio 1.6), younger age (odds ratio 1.02), and higher BMI (odds ratio 1.06).

Conclusions: We recommend AP with LAT images rather than either an AP or a LAT image alone.

## Introduction

Retained foreign body or surgical equipment can result in serious medical morbidities such as infection, pain, abscess and contribute to medicolegal problems [1,2]. The most common type of retained foreign body is gauze sponges, which can be referred to as gossypiboma, textiloma, gauzoma, or muslinoma [3,4]. Many institutes have different protocols on counting surgical instruments, but this adverse event still continues to happen in the surgical world. The real world incidence is difficult to estimate but incidence of retained sponges have been quoted from 1/1000 to 1/5500 [5-7]. Most of the data comes from the either abdominal surgeries or from insurance company databases.

When a retained surgical sponge is suspected, intraoperative X-ray images can be obtained to rule out a foreign body [1]. One radiographic image of the surgical field is most commonly preferred. However, a retained surgical sponge may not appear on intraoperative X-rays. Currently, there is no clinic study in the literature which reports the efficacy of the use of intraoperative radiographs to detect a retained sponge.

Revesz et al. looked at six different types and sizes of surgical sponges at laminectomy sites in a cadaver study [8]. They concluded that accuracy detecting sponges did not vary significantly, regardless of level of training or field of study. However, they did not analyze gauze sponges which are usually used in the lateral gutter alongside the transverse processes. The aim of the current study is to compare the sensitivity and specificity of intraoperative X-rays in cases of open posterior lumbar spine surgeries.

## Materials and methods

Study design

This was a prospective, randomized study. Anteroposterior (AP) and lateral (LAT) radiographs are standard of care during spine surgery to confirm proper implant placement. These images were used in this study. In one-half of the study subjects, after all sponges were removed, a surgical sponge was intentionally placed in the surgical field prior to imaging. Sponges were 4"x4" AMD-Ritmed Gauze Sponges (AMD Medicom, Inc., Montreal, Canada), 16-ply, 100% cotton with 2 zigzag X-ray markers (barium sulfate ≥ 55% and PVC base material). Postoperatively, each subject’s images were assessed for the presence or absence of a retained surgical sponge. De-identified images were randomly assigned to and read by two investigators.

This was a semi-blinded trial. Investigators performing the surgery knew if a sponge was present but the investigators reviewing the images did not. Subjects did not know their cohort assignment. Randomization was performed prior to surgery using sealed envelopes. For each of the subjects who had consented, when surgery was scheduled, an envelope was opened and the surgery worksheet was annotated, as appropriate. One co-investigator kept track of subject identifiers, subject numbers, and randomization.

Subject consent and inclusion

This study was approved by our local institutional review board before the study commenced (Allina Health Institutional Review Board, Reference Number 1290533-15). The trial registration number was NCT040976678. All subjects provided written informed consent to our use of their medical records and images for research. The inclusion criterion was patients undergoing open posterior instrumented lumbar spine surgery. The exclusion criteria were 1) patients who were pregnant, 2) patients who did not consent to research, 3) patients who were less than 18 years old at the time of consent, and 4) patients who did not read and understand English.

Data collection

Demographic information included sex, age, and body mass index (BMI). Surgical information included the number of levels decompressed and instrumented and the type and configuration of instrumentation. Radiographic images included AP and LAT views of the surgical field prior to closing.

Intraoperative methods

For subjects in study group one, the following steps were observed. First, the operating room (OR) staff removed and counted all surgical sponges. Then, just prior to imaging, the surgeon purposely placed a sponge in the wound and stated this to the circulating nurse and scrub tech. AP and LAT radiographic images were taken of the surgical field. After imaging, the surgeon removed the sponge and stated that he removed it. The scrub tech confirmed that the sponge was removed; the circulating nurse noted this in the chart. Finally, the OR staff re-counted the surgical sponges. For subjects in study group two, the OR staff removed and counted all surgical sponges. Then, AP and LAT radiographic images were taken of the surgical field. Lastly, the OR staff re-counted the surgical sponges.

Image analysis

AP and LAT radiographs obtained in the OR were stored in the hospital's picture archiving and communication (PAC) system. Sometime after the surgery was complete, the X-rays were "pushed" to our local PAC system (Synapse PACS, Fujifilm Healthcare Solutions, Tokyo, Japan). Each image was opened, anonymized, exported as a Joint Photographic Experts Group (JPEG) file, and saved with the best image quality. The file name was completely independent of the patient's medical record. The image was viewed with Microsoft Photos (Microsoft Corporation, Redmond, Washington, United States). The observer recorded his impression concerning the presence or absence of a retained sponge in a spreadsheet, which had no links to patient identifiers, other observers, or other views of the same image. One investigator (who was not an observer) compiled the observers' impressions for statistical analysis.

Statistics

This study quantified the accuracy, reproducibility, sensitivity, and specificity of 1) an AP image alone, 2) a LAT image alone, and 3) AP and LAT images together for identifying the presence or absence of a surgical sponge. Accuracy and reproducibility were assessed using Cohen’s kappa statistic. Kappa values 93-100% were interpreted as excellent, 81-92% were very good, 61-80% were good, 41-60% were fair, 21-40% were slight, and 1-20% were poor [9,10]. We defined sensitivity as the probability of seeing a sponge when a sponge was present and specificity as the probability of not seeing a sponge when no sponge was present. The positive predictive value (PPV) is the percent of all positive tests that are true positives. Similarly, the negative predictive value (NPV) is the percent of all negative tests that are true negatives. We used the following definitions: true positive - sponge observed when sponge present; false positive - sponge observed when sponge absent; false negative - sponge not observed when sponge present; and true negative - sponge not observed when sponge absent. Our hypothesis for statistical analysis was that there would be 90% specificity and 90% sensitivity [8]. Our sample size (n=146) was chosen to calculate Cohen’s kappa statistic and is like that of Revesz et al., who analyzed 72 radiographs with sponges and 72 radiographs without sponges and reported false-positive and false-negative rates of 10% [8,11]. The results were tested for the effects of demographic and surgical variables using logistic regression analysis. Throughout, a p-value of 0.05 was considered significant. The data analysis for this paper was generated using the Real Statistics Resource Pack software (Release 7.6). Copyright (2013 - 2021) Charles Zaiontz. www.real-statistics.com.

## Results

We recruited 146 patients into the study; radiographs were not obtained for 35 subjects due to the intraoperative decisions unrelated to the study; 111 subjects were included in the analyses. Two fellowship-trained, board-eligible, orthopedic spine surgeons observed the images. There were 56 subjects in the Sponge cohort and 55 subjects in the No Sponge cohort. There were 50 females and 61 males (Table 1). On average, they were 63±11 years old at the time of surgery. Their median BMI was 30.0 (range, 17.8-53.7). The median number of instrumented levels was 2 (range, 1-9) (Table 2). Seventeen patients received only posterior instrumentation (pedicle screws and rods); 93 subjects received posterior instrumentation and interbody spacers; one subject received posterior instrumentation, interbody spacers, and an anterior L5-S1 lumbar plate.

**Table 1:** Patient demographics BMI: body mass index

Factor	Sponge (n=56)	No Sponge (n=55)	p-value
Sex (male:female)	32:24	29:26	0.64
Age at surgery (mean ± 1 SD)	63 ± 11	63 ± 11	0.92
BMI (median, range)	30.4 (19.5-46.0)	29.4 (17.8-53.7)	0.67

**Table 2:** Surgical factors PEEK: polyetheretherketone; Ti: titanium; IBD: interbody device; ALP: anterior lumbar plate

Factor	Sponge (n=56)	No Sponge (n=55)	p-value
Number of instrumented levels (median, range)	2 (1-8)	2 (1-9)	0.56
Instrumentation configuration (patients (n))			0.60
Pedicle screws/rods alone	9	9	
Pedicle screws/rods + PEEK IBD	42	40	
Pedicle screws/rods + Ti IBD	3	5	
Pedicle screws/rods + PEEK IBD + Ti IBD	1	2	
Pedicle screws/rods + Ti IBD + ALP	1	0	

Accuracy

**Figure 1:**
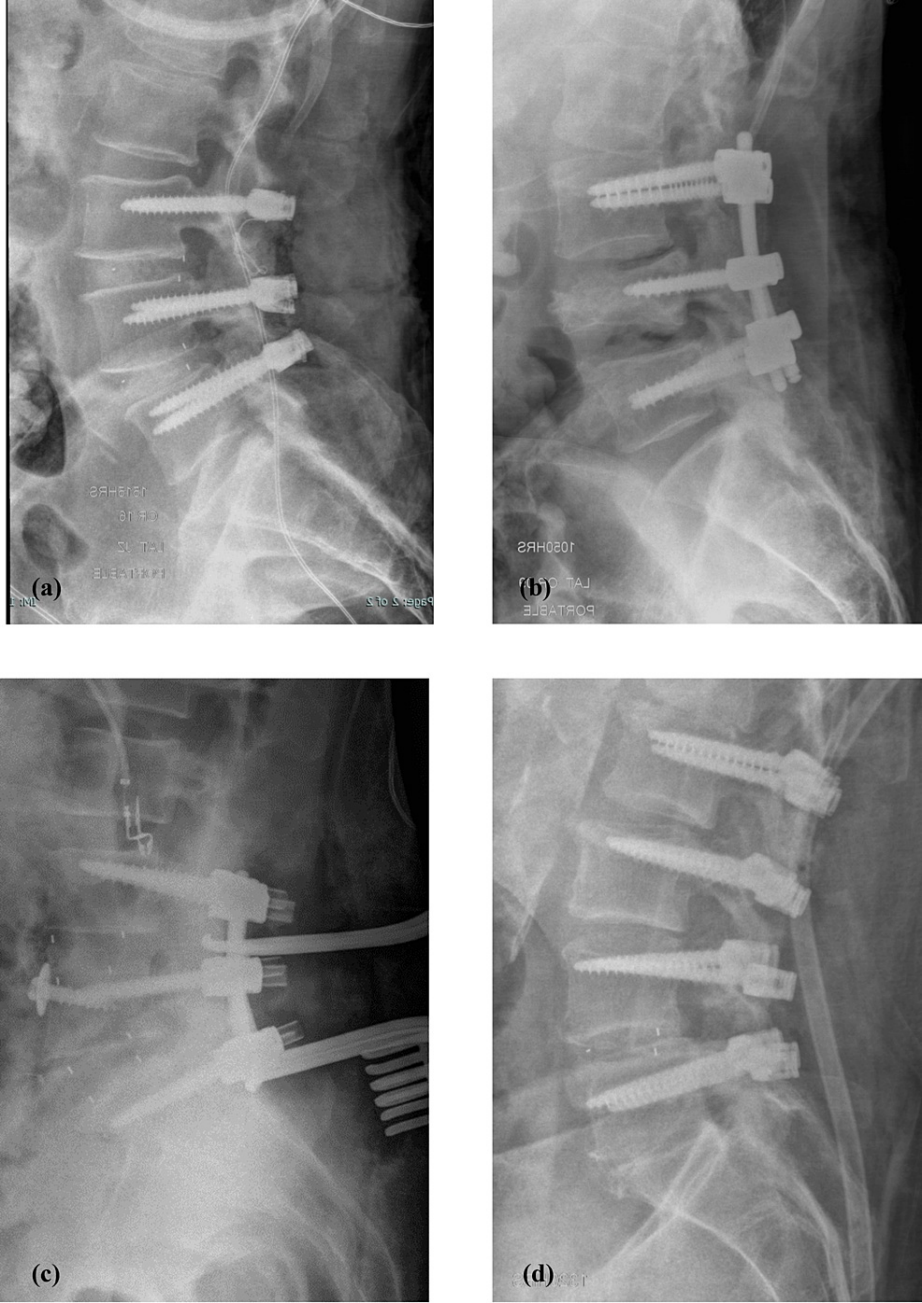
Lateral (LAT) radiographs - examples of (a) true positive (subject 1), (b) false negative (subject 4), (c) false positive (subject 113), and (d) true negative (subject 2)

**Figure 2:**
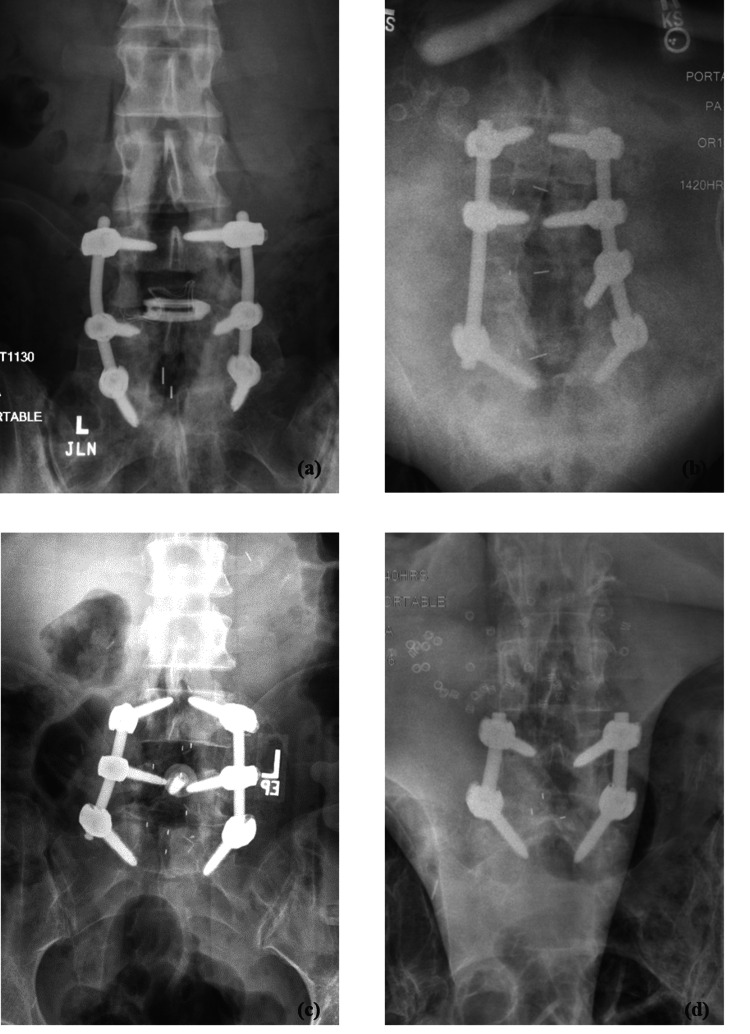
Anteroposterior (AP) radiographs - examples of (a) true positive (subject 65), (b) false negative (subject 7), (c) false positive (subject 113), (d) true negative (subject 3)

**Figure 3:**
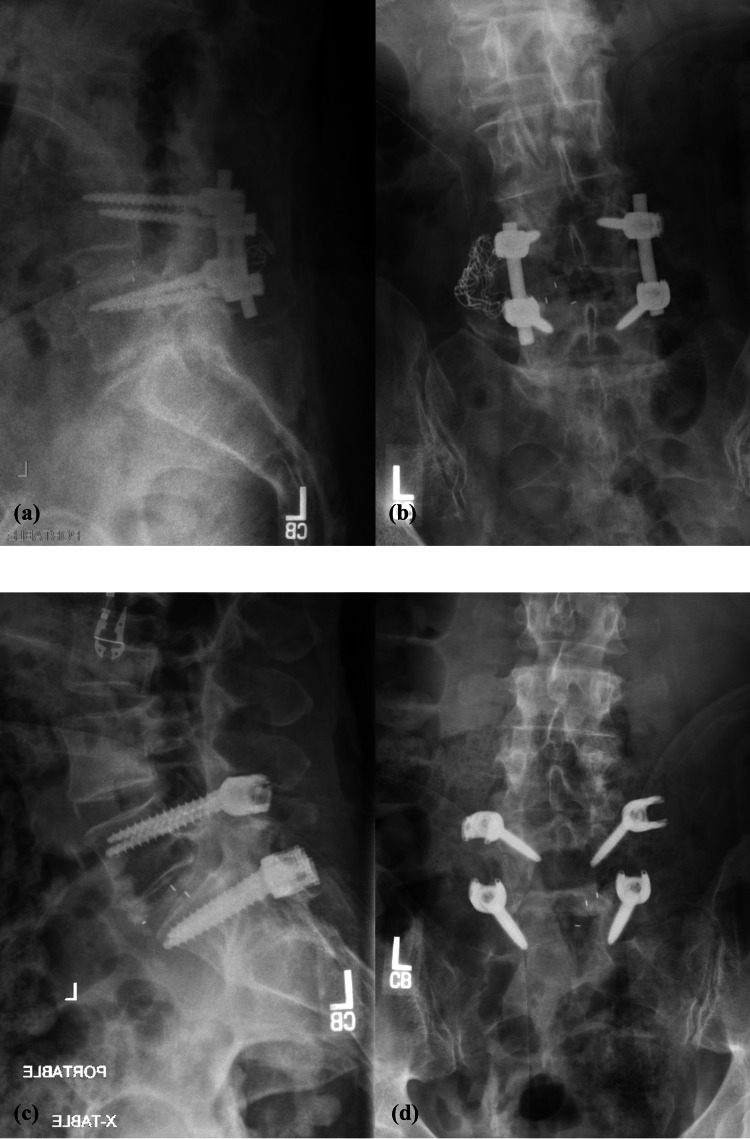
Lateral plus anteroposterior (LAT+AP) radiographs - examples of (a) true positive, LAT (subject 9); (b) true positive, AP (subject 9); (c) false negative, LAT (subject 14); and (d) false negative, AP (subject 14)

**Figure 4:**
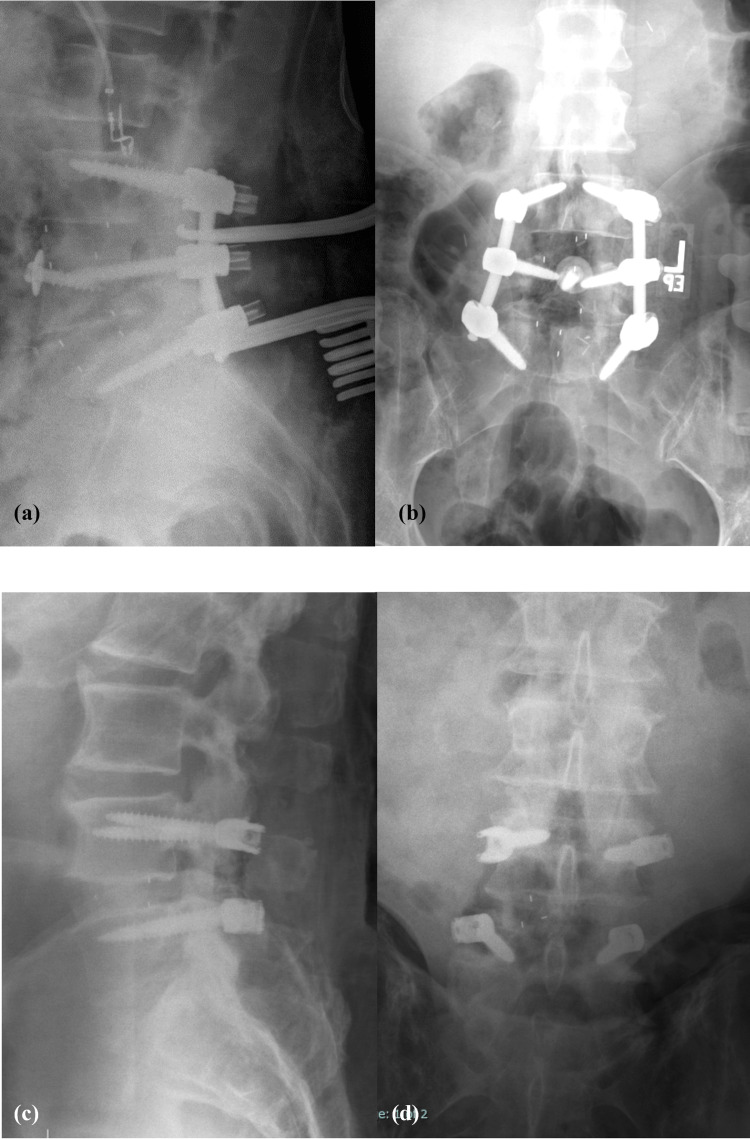
Lateral plus anteroposterior (LAT+AP) radiographs - examples of (a) false positive, LAT (subject 113); (b) false positive, AP (subject 113); (c) true negative, LAT (subject 8); and (d) true negative, AP (subject 8)

The agreement between the true diagnosis and the overall readers’ diagnoses, as assessed using Cohen’s kappa statistic, was 76% (good) for LAT images, 73% (good) for AP images, and 80% (good) for LAT+AP images (Table 3). Figures 1-4 depict a true positive, a true negative, a false positive, and a false negative for LAT, AP, and LAT+AP.

**Table 3:** Accuracy of radiographic assessment LAT: lateral; AP: anteroposterior; LAT+AP: lateral and anteroposterior; CI: confidence interval

LAT view		Cohen's kappa	Standard error	95% CI
Observer 1	1st Pass	0.71	0.07	0.58 - 0.84
	2nd Pass	0.73	0.07	0.60 - 0.85
	Average	0.72	0.07	0.59 - 0.85
Observer 2	1st Pass	0.80	0.06	0.69 - 0.91
	2nd Pass	0.82	0.05	0.71 - 0.93
	Average	0.81	0.06	0.70 - 0.92
Overall average		0.76	0.06	0.65 - 0.88
AP view		Cohen's kappa	Standard error	95% CI
Observer 1	1st Pass	0.73	0.07	0.60 - 0.86
	2nd Pass	0.61	0.08	0.46 - 0.76
	Average	0.67	0.07	0.53 - 0.81
Observer 2	1st Pass	0.80	0.06	0.69 - 0.91
	2nd Pass	0.80	0.06	0.69 - 0.91
	Average	0.80	0.06	0.69 - 0.91
Overall Average		0.73	0.06	0.61 - 0.86
LAT +AP views		Cohen's kappa	Standard error	95% CI
Observer 1	1st Pass	0.75	0.06	0.62 - 0.87
	2nd Pass	0.82	0.05	0.71 - 0.93
	Average	0.78	0.06	0.67 - 0.90
Observer 2	1st Pass	0.82	0.05	0.71 - 0.93
	2nd Pass	0.82	0.05	0.71 - 0.93
	Average	0.82	0.05	0.71 - 0.93
Overall average		0.80	0.06	0.69 - 0.91

Accuracy and patient factors

We performed a logistic regression on the pooled outcomes of the readers' diagnoses (right or wrong) for all patients, observers, readings, and views. We treated outcome as the dependent variable and patient age, sex, and BMI as independent variables. Age, sex, and BMI were statistically significant (Table 4). The probability of being right was better for female sex (odds ratio: 1.6), younger age (odds ratio: 1.02), and higher BMI (odds ratio: 1.06). However, age and BMI contributed little to explaining the variance because their beta coefficients are very small.

**Table 4:** Results of regression analysis for outcome accuracy* *A correct outcome by the observer was assigned the value of 1; an incorrect outcome was 0 †Male sex was assigned the value of 1; female sex was assigned the value of 2

Factor	Beta coefficient	Standard error	Wald statistic	p-value	Odds ratio	95% confidence interval	
						Lower bound	Upper bound
Intercept	0.516	0.777	0.441	0.507	1.675		
Sex†	0.448	0.176	6.468	0.011	1.565	1.108	2.210
Age	-0.016	0.008	4.148	0.042	0.984	0.969	0.999
BMI	0.061	0.015	16.847	0.000	1.063	1.032	1.094

Interobserver reproducibility

The agreement between the observers was 89% (very good) for LAT images, 85% (very good) for AP images, and 96% (excellent) for LAT+AP images (Table 5).

**Table 5:** Interobserver reproducibility of radiographic assessment LAT: lateral; AP: anteroposterior; LAT+AP: lateral and anteroposterior

LAT view		Cohen's kappa
Observer 1 versus Observer 2	1st Pass	0.87
	2nd Pass	0.91
	Average	0.89
AP View		Cohen's kappa
Observer 1 versus Observer 2	1st Pass	0.93
	2nd Pass	0.78
	Average	0.85
LAT+AP views		Cohen's kappa
Observer 1 versus Observer 2	1st Pass	0.93
	2nd Pass	1.00
	Average	0.96

Intraobserver reproducibility

The agreement of the observer with himself was 91% (very good) for LAT images, 88% (very good) for AP images, and 96% (excellent) for LAT+AP images (Table 6).

**Table 6:** Intraobserver reproducibility of radiographic assessment LAT: lateral; AP: anteroposterior; LAT+AP: lateral and anteroposterior

LAT view	Cohen's kappa
Observer 1, 1st versus 2nd pass	0.83
Observer 2, 1st versus 2nd pass	0.98
Average	0.91
AP view	Cohen's kappa
Observer 1, 1st versus 2nd pass	0.78
Observer 2, 1st versus 2nd pass	0.98
Average	0.88
LAT+AP views	Cohen's kappa
Observer 1, 1st versus 2nd pass	0.93
Observer 2, 1st versus 2nd pass	1.00
Average	0.96

Sensitivity and specificity

The average sensitivity (the percent of all positive tests that are true positives) was 82% (very good) for LAT images, 86% (very good) for AP images, and 87% (very good) for LAT+AP images (Table 7). The specificity (the percent of all negative tests that are true negatives) was 95% (excellent) for LAT images, 88% (very good) for AP images, and 93% (excellent) for LAT+AP images.

**Table 7:** Sensitivity and specificity of radiographic assessments LAT: lateral; AP: anteroposterior; LAT+AP: lateral and anteroposterior

LAT view		Sensitivity	Specificity
Observer 1	1st Pass	77%	94%
	2nd Pass	80%	92%
	Average	79%	93%
Observer 2	1st Pass	84%	96%
	2nd Pass	86%	96%
	Average	85%	96%
Overall average		82%	95%
AP view		Sensitivity	Specificity
Observer 1	1st Pass	84%	89%
	2nd Pass	87%	74%
	Average	86%	81%
Observer 2	1st Pass	86%	95%
	2nd Pass	85%	95%
	Average	86%	95%
Overall average		86%	88%
LAT+AP views		Sensitivity	Specificity
Observer 1	1st Pass	88%	87%
	2nd Pass	88%	94%
	Average	88%	91%
Observer 2	1st Pass	88%	95%
	2nd Pass	87%	95%
	Average	87%	95%
Overall average		87%	93%

PPV and NPV

The average PPV (the percent of all positive readings that are true positives) was 94% (excellent) for LAT images, 89% (very good) for AP images, and 93% (excellent) for LAT+AP images (Table 8). The average NPV (the percent of all negative readings that are true negatives) was 83% (very good) for LAT images, 86% (very good) for AP images, and 88% (very good) for LAT+AP images.

**Table 8:** PPV and NPV of radiographic assessments LAT: lateral; AP: anteroposterior; LAT+AP: lateral and anteroposterior; PPV: positive predictive value; NPV: negative predictive value

LAT view		PPV	NPV
Observer 1	1st Pass	93%	80%
	2nd Pass	92%	82%
	Average	93%	81%
Observer 2	1st Pass	96%	85%
	2nd Pass	96%	87%
	Average	96%	86%
Overall average		94%	83%
AP view		PPV	NPV
Observer 1	1st Pass	89%	84%
	2nd Pass	77%	85%
	Average	83%	84%
Observer 2	1st Pass	94%	87%
	2nd Pass	94%	87%
	Average	94%	87%
Overall average		89%	86%
LAT+AP views		PPV	NPV
Observer 1	1st Pass	88%	87%
	2nd Pass	94%	88%
	Average	91%	87%
Observer 2	1st Pass	94%	88%
	2nd Pass	94%	88%
	Average	94%	88%
Overall average		93%	88%

## Discussion

From the few cases that have been reported in the literature, the most commonly retained foreign body has been sterile sponges, often used for hemostasis [1]. Most hospitals have protocols to follow if the surgical counts do not add up. These may include recounting and searching of the foreign body on the floor and in the drapes. If this fails to find the missing foreign body, the next step usually is to have intraoperative radiographs, which are analyzed by the surgeon and confirmed by a radiologist in order for wound closure [8,12]. Currently, there is no clinical study in the spine literature that has studied the specificity and sensitivity of intraoperative radiographs to detect retained sponges. The aim of the current study is to compare the sensitivity and specificity of intraoperative X-rays in cases of open posterior lumbar spine surgeries.

Radiographs are the most utilized method to detect retained sponges. This is possible because most sponges will have embedded radiopaque markers. Intraoperative or portable early postoperative radiographs may be of suboptimal quality, and hot lighting of hard-copy radiographs or digital magnification and manipulation of soft-copy images are recommended [12]. Folded and twisted sponges and their markers can be difficult to recognize, even by radiologists [13,14]. Postoperatively, if radiographs do not identify the missing foreign body, a CT scan or MRI has been shown to be effective [1]. But from a practical standpoint, prior to closure of the wound, AP and LAT radiographs appear to be the best method to detect retained sponges.

In the current study, Cohen’s kappa statistics were “good” to “excellent” (Table 9). Based on these findings, we recommend using LAT+AP images to decide if a sponge is or is not present, instead of just one radiograph, AP or LAT.

**Table 9:** Summary of Cohen’s kappa statistics *Best among the three views LAT: lateral; AP: anteroposterior; LAT+AP: lateral and anteroposterior: PPV: positive predictive value; NPV: negative predictive value

Measure	LAT view	AP view	LAT+AP views
Accuracy	Good	Good	Good*
Interobserver agreement	Very Good	Very Good	Excellent*
Intraobserver agreement	Very Good	Very Good	Excellent*
Sensitivity	Very Good	Very Good	Very Good*
Specificity	Excellent*	Very Good	Excellent
PPV	Excellent*	Very Good	Excellent
NPV	Very Good	Very Good	Very Good*

It was an interesting finding in the current study that the probability of being right was better for female sex (odds ratio: 1.6), younger age (odds ratio: 1.02), and higher BMI (odds ratio: 1.06). Although the beta coefficient was small, it was counterintuitive to the general belief that a higher BMI would make it difficult to identify sponges on radiographs. The regression analysis also suggests that special attention should be given to older male patients.

One strength of the current study is that it is a prospective randomized semi-blind study. Another strength is that the analyses were performed by two fellowship-trained spine surgeons using an easily reproducible method. A study limitation is our use of anonymized JPEG images, which were exported from our local PAC system, rather than the original images in the PAC system. Another limitation is that we studied only one scenario, namely that of one sponge placed in the lateral gutter; other locations, other objects, other approaches, and other regions of the spine (e.g., the neck) were not studied.

## Conclusions

A retained foreign object can create serious medical morbidities after surgery, and the most commonly retained foreign object is gauze sponges. Intraoperative X-ray images are routinely used to locate a missing sponge, but it may or may not appear on the image. Currently, there is no published clinical study reporting the efficacy of intraoperative radiographs in detecting a retained sponge. Therefore, we analyzed the accuracy of radiographs to detect retained sponges in cases of open posterior spine surgeries. As such, it provides support to a practice that lacked data. While intraoperative images are often read by radiologists prior to the closure of the wound, they are first seen and interpreted by the operating surgeon. The premise of our study was to identify the presence/absence of retained sponges by the operating surgeon, who is ultimately responsible and by whom decisions are made in the operating room. Overall, we found it best when LAT and AP images were used to decide if a sponge was or was not present, instead of just one lateral or anteroposterior radiograph. Our results should give confidence to surgeons when facing the difficult clinical problem of a missing sponge.
